# Greedy Successive Anchorization for Localizing Machine Type Communication Devices

**DOI:** 10.3390/s16122115

**Published:** 2016-12-13

**Authors:** Mian Imtiaz Ul Haq, Dongwoo Kim

**Affiliations:** Department of Electronics and Communication Engineering, Hanyang University, Ansan 15588, Korea; imtiaz@hanyang.ac.kr

**Keywords:** distributed localization, successive anchorization, positioning error, Cramér–Rao lower bound

## Abstract

Localization of machine type communication (MTC) devices is essential for various types of location-based applications. In this paper, we investigate a distributed localization problem in noisy networks, where an estimated position of blind MTC machines (BMs) is obtained by using noisy measurements of distance between BM and anchor machines (AMs). We allow positioned BMs also to work as anchors that are referred to as virtual AMs (VAMs) in this paper. VAMs usually have greater position errors than (original) AMs, and, if used as anchors, the error propagates through the whole network. However, VAMs are necessary, especially when many BMs are distributed in a large area with an insufficient number of AMs. To overcome the error propagation, we propose a greedy successive anchorization process (GSAP). A round of GSAP consists of consecutive two steps. In the first step, a greedy selection of anchors among AMs and VAMs is done by which GSAP considers only those three anchors that possibly pertain to the localization accuracy. In the second step, each BM that can select three anchors in its neighbor determines its location with a proposed distributed localization algorithm. Iterative rounds of GSAP terminate when every BM in the network finds its location. To examine the performance of GSAP, a root mean square error (RMSE) metric is used and the corresponding Cramér–Rao lower bound (CRLB) is provided. By numerical investigation, RMSE performance of GSAP is shown to be better than existing localization methods with and without an anchor selection method and mostly close to the CRLB.

## 1. Introduction

Machine type communication (MTC) services with location-based information are becoming popular such as combat zone surveillance, health monitoring, fire detection and wild habitat monitoring [1,2,3]. MTC devices (in this paper, we use the term “devices” and “machines” interchangeably) with which sensors are equipped as a basic component are usually distributed randomly, accessed from far-away and configured automatically. Thus, location information in MTC services is crucial. One way to get position information is to fit each MTC device with global positioning system (GPS) that enables it to get its position by interacting with GPS satellites. However, this method may not be cost-effective for large scale implementation and is not even possible for indoor or space applications, where MTC devices cannot receive GPS signals [4].

When GPS is not available, each MTC device will estimate its location based on its relative distances to some of the other MTC devices that already have acquired their positions, which are so-called anchor machines (AMs). Localization algorithms that are used to determine the unknown location of devices are computationally complicated in radar and sonar systems [5,6]. However, MTC devices are usually small, and, hence, the localization algorithms should be simple and distributed [7,8,9,10,11].

In [7], a distributed and range-free centroid localization (CL) method is proposed on the assumption that every blind machine (BM that needs to determine its location) should be in the neighbor of three or more AMs. The CL is extremely simple but a little bit inaccurate and requires uniformly distributed AMs near BM. Weighted centroid localization (WCL) improves CL by giving different weights to AMs on the basis of noisy distance information between BM and AMs [8]. WCL gives more importance to the nearer AMs. However, giving how much or finding an optimal weight is the underlying difficulty. In [9], computing an optimal weight in WCL is considered with received signal strength (RSS), which is effective only when AMs are densely distributed.

In practice, it is not easy for blind MTC devices to hear from a sufficient number of AMs since the range of MTC communication is usually limited due to the battery capacity. To overcome this problem, we investigate a method of using BMs that have determined their positions as new anchors, referred to as virtual AMs (VAMs) in this paper. Compared with ordinary (original) AMs, VAMs could have relatively high position errors but if no AM is found (for example, in indoor environments), using VAMs to determine the location BM is acceptable. Though AM itself can have position error, most of the works in this field have assumed that the location information is perfectly accurate [12,13]. However, in a few works [14,15], the position error in AMs is modeled as Gaussian noise and a gradual refinement of the localization error is also proposed [16]. In this paper, we assume that every VAM estimates its position error during its localization process and broadcasts the estimation on its beacon. The position error in VAMs is due to the noise in measurement as well as the error in the location of AMs used in determining its location. In the localization method proposed in this paper, BM can change into and be used as VAM after determining its position, which is referred to as a *successive anchorization process* (SAP). With SAP, the localization errors propagate through the whole network. One of the objectives of this paper is to propose an efficient localization method that minimizes the localization errors over the whole MTC network. To achieve this goal, every BM chooses VAMs (usually three VAMs are enough) with the smallest localization error in the proposed algorithm, which is referred to as a *greedy* SAP (GSAP).

In order to speed up the computation for GSAP through the network, each BM takes a linearization of its non-linear distance error-function by using the first-order Taylor expansion. The proposed GSAP is essentially distributed in a sense that each BM determines its location only with the location information of AMs or VAMs from its neighbour, starting from an arbitrary initial position. To have fast convergence of distributed GSAP, a decentralized method of finding a good initial position is provided, which is based on a multidimensional scaling (MDS) technique [17]. Using a good initial position is, however, optional in implementing GSAP. We numerically investigate the effect of using this good initial position in GSAP. The Cramér–Rao lower bound (CRLB) of the overall error is also investigated and the performance of the proposed method is numerically compared with CRLB and other existing methods such as CL, WCL, LLS (linear least square) [10] and the subspace technique [11]. The results show that the proposed GSAP significantly improves the BM position accuracy, and it can also attain the performance mostly close to CRLB.

The rest of the paper is organized as follows. In Section 2, a system model used in this paper is provided. Section 3 provides GSAP that consists of a greedy anchor selection, a linearized iterative localization algorithm and (optional) initial position search. In Section 4, CRLB of the overall position error is investigated. In Section 5, numerical investigation is provided for evaluating the proposed GSAP, for which the root mean square error (RMSE) results of existing localization methods are compared with that of GSAP. Section 6 concludes the paper. The main notations used in this paper are listed in Table 1.

## 2. System Model

We consider an MTC network that consists of *n* BMs and *m* AMs or VAMs in a two-dimensional space (this work also can be straightforwardly applied for localization in a three-dimensional space). Let N=n+m and $\phi_{i}={\left[x_{i},y_{i}\right]}^{T}\left(\in R^{2}\right)$ denote the actual coordinates of machine *i*, and let $S_{A}$, $S_{V}$ and $S_{U}$ be index sets of AMs, VAMs and BMs, respectively. We assume that each AM or VAM knows its position as well as its positioning error $\vartheta_{j}^{p}$ ($j\in S_{A}\cup S_{V}$). It is reasonable to assume that the positioning error of AM is less than or at least equal to that of VAM.

In the MTC environments, each machine is capable of communication with the other machines if they are in the communication range. We use RSS measurement to determine unknown positions of BMs. We assume that each AM and VAM broadcasts reference signals as well as its coordinates with the positioning error (and sometimes with a value of its transmit power). BMs that can hear the information from three or more anchors first select the anchor nodes and then run a localization algorithm to be provided below. It is noted that the localization algorithm is distributed in nature, that is, each BM independently determines its position without cooperation by the other BMs.

We assume that transmit power (denoted by $P_{j},j\in S_{V}\cup S_{A}$) for the broadcast information is known to BMs. Let $P_{ij}$ denote RSS measurement at BM *i* from anchor *j*. Then, we can use a path loss model in [18]:

(1) $$\begin{array}{ccc} P_{ij}=P_{j}+\varsigma_{ij}\left(\phi \right)+\eta_{ij}, & & i\in S_{U},j\in S_{V}\cup S_{A}, \end{array}$$

where $\varsigma_{ij}\left(\phi \right)=-10\beta \log d_{ij}$, *β* is a distance–power gradient (i.e., a path loss exponent), $d_{ij}$ is the actual distance between machines *i* and *j*, and $\eta_{ij}$ is noisy power due to measurement errors at BM *i* and unmodeled variability in the fading channel between machines *i* and *j* (for example, shadowing). We assume that $\eta_{ij}$s are independently distributed zero-mean Gaussian random variables with standard deviation $\sigma_{ij}$ (dB). We also assume that $\sigma_{ij}$ is known to BMs. From the RSS measurements, each BM can compute the expected error in measurement denoted by $\vartheta_{ij}^{r}$ $\left(i\in S_{U},j\in S_{V}\cup S_{A}\right)$. If BM *i* receives *L* RSS samples from anchor *j*, $\vartheta_{ij}^{r}=10^{\sigma_{ij}/10}/L$ since noisy power is assumed to be independent.

## 3. Greedy Successive Anchorization Process (GSAP)

### 3.1. Anchor Selection

#### 3.1.1. Greedy Selection

BM *i* selects anchors to be used in the localization process by comparing

(2) $$\begin{array}{c} \zeta_{ij}=\frac{1}{{\left(\vartheta_{j}^{p}\right)}^{-1}+{\left(\vartheta_{ij}^{r}\right)}^{-1}}, \end{array}$$

where *j* is an index of anchors in its communication range, from which the BM successfully receives the broadcast information. Since three anchors are enough to determine the location of BM in two-space, each BM selects anchors up to smallest $\zeta_{ij}$, which is referred to as *greedy selection* in this paper.

#### 3.1.2. Removing Collinear Anchors

If anchors selected from Section 3.1.1 are collinear, high localization error is caused mostly due to vertex flipping instead of the position or the measurement error [19,20]. In order to overcome such a problem, we test $a\;\sin^{2}\gamma >d_{min}$, where *a* and *γ* are the shortest side and the smallest angle of the triangle built by the greedy-selected three anchors, and $d_{min}$ is a predetermined constant. This test is provided in [19] as a method of constructing a so-called robust triangle. If the three anchors fail to pass the test, we select another anchor that has the next smallest $\zeta_{ij}$ and do the same test for every combination of the selected anchors. If the test fails for all the anchors whose information is available at a BM, the BM detours the localization procedure for the next round in which more anchors are possibly available since new VAMs can be added to the list of anchors.

#### 3.1.3. Other Anchor Selection Methods in the Literature

Though all of the existing anchor selection methods do not consider the position error at an anchor, there are some methods in the literature. In CL [7], BM selects all those anchors that are one-hop neighbors of that BM. In [21], in order to increase the accuracy, a convex-hull anchor selection method is proposed by considering the geometry of anchors, where only those anchors that have the greatest distance from each other are selected. When applying the convex-hull method with RSS measurement, possibly large measurement error is problematic since the increasing distance between anchors usually increases the distance between an anchor and BM, and thus the error [22]. The work of [21] has proposed an advanced convex-hull method where BM selects those AMs that are close to the ordinary convex-hull with the highest virtual location accuracy. The main drawback of this method is that it assumes highly dense anchors. We will numerically compare the performance of proposed greedy selection with that of the existing methods in Section 5.

### 3.2. Iterative Localization Algorithm

Let $S_{S}$ be a set of selected anchors and $p_{i}=\left\{p_{ij}=P_{ij}-P_{j},j\in S_{S}\right\}$ be a column vector of augmented RSS measurement (or observed path loss) at BM *i* from the selected anchors. BM *i* $\left(i\in S_{U}\right)$ determines (or estimates) its position ${\hat{\phi }}_{i}$ by

(3) $$\begin{array}{c} {\hat{\phi }}_{i}=\arg\min\Theta \left(\phi_{i}\right), \end{array}$$

where

(4) $$\begin{array}{c} \Theta \left(\phi_{i}\right)={\left[p_{i}-\varsigma \left(\phi_{i}\right)\right]}^{T}\left[p_{i}-\varsigma \left(\phi_{i}\right)\right]. \end{array}$$

Furthermore, $\varsigma (\phi_{i})$ is a column vector, the element of which is

(5) $$\begin{array}{c} \begin{array}{cccc} \varsigma_{j}\left(\phi_{i}\right) & =-5\beta [\log{∥\phi_{i}-\phi_{j}∥}^{2}], & & j\in S_{S}. \end{array} \end{array}$$

A general solution of Equations (3) and (4) can be obtained through a nonlinear optimization method [23,24,25], which iterates to get the optimal value in the feasible region. However, its computational complexity is usually very high [26]. In order to make the computational cost low [27], we linearize $\varsigma (\phi_{i})$ by using the first-order Taylor series expansion [28]

(6) $$\begin{array}{c} \varsigma \left(\phi_{i}\right)\approx \varsigma \left({\tilde{\phi }}_{i}\right)+\Pi \left(\phi_{i}-{\tilde{\phi }}_{i}\right), \end{array}$$

where

(7) $$\begin{array}{c} \Pi =-10\beta \left[\begin{array}{cc} \frac{\cos\alpha_{i1}}{∥\phi_{i}-\phi_{1}∥}{|}_{\phi_{i}={\tilde{\phi }}_{i}} & \frac{\sin\alpha_{i1}}{∥\phi_{i}-\phi_{1}∥}{|}_{\phi_{i}={\tilde{\phi }}_{i}}\\ \vdots & \vdots \\ \frac{\cos\alpha_{ij}}{∥\phi_{i}-\phi_{j}∥}{|}_{\phi_{i}={\tilde{\phi }}_{i}} & \frac{\sin\alpha_{ij}}{∥\phi_{i}-\phi_{j}∥}{|}_{\phi_{i}={\tilde{\phi }}_{i}} \end{array}\right] \end{array}$$

and ${\tilde{\phi }}_{i}$ is any initially estimated position of BM *i* (finding a good ${\tilde{\phi }}_{i}$ will be shortly discussed in Section 3.3). In the above expression and in the sequel, we assume that 1 to *j* are indexes of the selected anchors for notational brevity. Putting Equation (6) into Equation (4), we have

(8) $$\begin{array}{c} \Theta \left(\phi_{i}\right)\approx {\left[p_{i}-\varsigma \left({\tilde{\phi }}_{i}\right)-\Pi \left(\phi_{i}-{\tilde{\phi }}_{i}\right)\right]}^{T}\left[p_{i}-\varsigma \left({\tilde{\phi }}_{i}\right)-\Pi \left(\phi_{i}-{\tilde{\phi }}_{i}\right)\right]. \end{array}$$

In [29], it is shown that the localization accuracy can be increased by inserting a symmetric weighting matrix R into Equation (8), and the resulting equation is defined as weighted least square (WLS), which has the form

(9) $$\begin{array}{c} \Theta_{\left(\mathrm{WLS}\right)}\left(\phi_{i}\right)\approx {\left[p_{i}-\varsigma \left({\tilde{\phi }}_{i}\right)-\Pi \left(\phi_{i}-{\tilde{\phi }}_{i}\right)\right]}^{T}R\left[p_{i}-\varsigma \left({\tilde{\phi }}_{i}\right)-\Pi \left(\phi_{i}-{\tilde{\phi }}_{i}\right)\right]. \end{array}$$

Using Equation (9), an approximate solution of Equation (3) is obtained as

(10) $$\begin{array}{c} {\tilde{\phi }}_{i}^{*}={\left(\Pi^{T}R\Pi \right)}^{-1}\Pi^{T}R[p_{i}-\varsigma \left({\tilde{\phi }}_{i}\right)+\Pi {\tilde{\phi }}_{i}]. \end{array}$$

As a weighting matrix, we use $R=\mathrm{diag}\left[\zeta_{i1},\zeta_{i2},\cdots ,\zeta_{ij}\right]$, where $\zeta_{ij}$s are the metrics used in anchor selection in this paper.

The proposed method stops with a solution ${\tilde{\phi }}_{i}^{*}$ if $∥\Theta_{\left(\mathrm{WLS}\right)}({\tilde{\phi }}_{i}^{*})-\Theta_{\left(\mathrm{WLS}\right)}({\tilde{\phi }}_{i})∥<\delta$ for a given positive termination threshold *δ*. Otherwise, we update the initial estimate as ${\tilde{\phi }}_{i}={\tilde{\phi }}_{i}^{*}$ and find the next solution in Equation (10) again. At the termination, BM *i* can estimate its position error by $\vartheta^{p}=1/\mathrm{Tr}\left({\left(\Pi^{T}R\Pi \right)}^{-1}\right)$, which will be broadcast by BM *i* as a VAM after finding its position.

### 3.3. Initial Location Estimation

If BM has received information from a sufficient number of AMs or VAMs and starts to determine its location, it needs a certain initial estimate of its location when the linearization provided in Equation (6) is evoked. Though the proposed algorithm in Section 3.2 works well with any arbitrary initial point, we describe a method of finding a good initial point in the following and will show that it improves the convergence speed significantly in Section 5. The following method is based on MDS in [17] and implemented in a distributed way.

Let N−1 be the number of AMs or VAMs, the signals from which BM can hear, and let index 1 denote the current BM that wants to have its initial location. In addition, 2,3,⋯,N indicate the AMs and the VAMs for notational simplicity. Let ${\tilde{d}}_{ij}$ be the distance estimate between machine *i* and *j*. Since BM 1 can measure RSS from the anchors, we can have, from Equation (1),

(11) $$\begin{array}{c} {\tilde{d}}_{1j}\approx 10^{\frac{P_{j}-P_{1j}}{10\beta }},\;\;\mathrm{for}\;j=2,3,\cdots ,N. \end{array}$$

Since the BM can see the position of anchors in the broadcast information, letting $\left({\tilde{x}}_{j},{\tilde{y}}_{j}\right)$ be the broadcast coordinate from anchor *j*, it can estimate the distance between those anchors by

(12) $$\begin{array}{c} {\tilde{d}}_{ij}={\left[{\left({\tilde{x}}_{i}-{\tilde{x}}_{j}\right)}^{2}+{\left({\tilde{y}}_{i}-{\tilde{y}}_{j}\right)}^{2}\right]}^{0.5}\;\;\mathrm{for}\;i,j=2,3,\cdots ,N\;\mathrm{and}\;i\neq j. \end{array}$$

If we further let ${\tilde{d}}_{jj}=0$, then we can construct a so-called proximity information matrix (PIM) D = ${\left[D\right]}_{ij}={\tilde{d}}_{ij}^{2}$. It is noted that D is an N×N square symmetric matrix.

Using PIM D, an objective of MDS is to find relative coordinates $\tilde{\Phi }$ of the machines in the hearing range of BM 1, such that $d_{ij}\left(\tilde{\Phi }\right)=∥{\tilde{\phi }}_{i}-{\tilde{\phi }}_{j}∥$ matches ${\tilde{d}}_{ij}$ as well as possible. For this purpose, an optimization problem to be solved is

(13) $$\begin{array}{c} \tilde{\Phi }=\arg\min_{\Phi }X_{D}\left(\Phi \right), \end{array}$$

where

(14) $$\begin{array}{c} X_{D}\left(\Phi \right)=\frac{\sqrt{\sum_{i=1}^{N}\sum_{j>i}{\left({\tilde{d}}_{ij}-d_{ij}\left(\tilde{\Phi }\right)\right)}^{2}}}{\sum_{i=1}^{N}\sum_{j>i}{\tilde{d}}_{ij}} \end{array}$$

is a so-called stress function provided in [30]. A solution is obtained by decomposing a double centered matrix **Ω** so as to have $\Omega \overset{\Delta }{=}{\tilde{\Phi }}^{T}\tilde{\Phi }$ [31], where each element of **Ω** is constructed by

(15) $$\begin{array}{cc} \omega_{ij} & =-0.5\left(\frac{1}{N^{2}}\sum_{i=1}^{N}\sum_{j=1}^{N}{\tilde{d}}_{ij}^{2}-\frac{1}{N}\sum_{i=1}^{N}{\tilde{d}}_{ij}^{2}+{\tilde{d}}_{ij}^{2}-\frac{1}{N}\sum_{j=1}^{N}{\tilde{d}}_{ij}^{2}\right). \end{array}$$

It is noted that the rank of **Ω** is 2. Let $\Lambda =\mathrm{diag}(\lambda_{1},\lambda_{2},\cdots ,\lambda_{N})$ be a diagonal matrix of the eigenvalue $\lambda_{j}$s of **Ω**. In addition, let $C=[c_{1},c_{2},\ldots ,c_{N}]$ be a matrix of the eigenvectors $c_{j}$ corresponding to $\lambda_{j}$. Then, taking eigendecomposition of **Ω** gives $\Omega =C\Lambda C^{T}$, and we have a solution

(16) $$\begin{array}{c} \tilde{\Phi }=C\Lambda^{0.5}={\left\{{\tilde{\phi }}_{j}\right\}}_{j=1}^{N} \end{array}$$

and can use ${\tilde{\phi }}_{1}$ as an initial position of BM.

## 4. Cramér–Rao Lower Bound

In this section, we derive a CRLB of the localization error for multiple $n_{b}$ BMs that commonly use *m* AMs and $n_{v}$ VAMs. Let $\phi_{b}$ denote the position vector of BMs. We assume that AMs have their locations without position error and without loss of generality. Let us denote actual and estimated positions of VAMs by $\phi_{v}$ and ${\hat{\phi }}_{v}$, respectively. The errors in measurement p as well as position ${\hat{\phi }}_{v}$ are Gaussian-distributed and mutually independent. The log-likelihood function of data vector $x={\left[p^{T},{\hat{\phi }}_{v}^{T}\right]}^{T}$ is then

(17) $$\begin{array}{c} \begin{array}{cc} L(x;\varrho ) & =\ln g\left(p;\varrho \right)+\ln g\left({\hat{\phi }}_{v};\varrho \right)\\ & =K-0.5\left\{\sum_{j=1}^{a}\sum_{i=n_{v}+1}^{n_{b}}\frac{1}{\sigma_{ij}^{2}}{\left(p_{ij}-\varsigma_{ij}\left(\phi \right)\right)}^{2}+{\left({\hat{\phi }}_{v}-\phi_{v}\right)}^{T}Q_{\phi_{v}}^{-1}\left({\hat{\phi }}_{v}-\phi_{v}\right)\right\}, \end{array} \end{array}$$

where $\varrho ={\left[\phi_{b}^{T},\phi_{v}^{T}\right]}^{T}$ represents the unknown vector, K is a constant that is independent of the unknowns, and $Q_{\phi_{v}}$ is a covariance matrix of position errors in ${\hat{\phi }}_{v}$.

Let $\hat{\varrho }=\hat{\varrho }\left(x\right)$ denote a position estimator. Then, the error performance can be determined by its bias $b\left(\hat{\varrho }\right)=E\left[\hat{\varrho }\right]-\varrho$, its covariance $℧\left(\hat{\varrho }\right)=E[\left(\hat{\varrho }-E\left[\hat{\varrho }\right]\right){\left(\hat{\varrho }-E\left[\hat{\varrho }\right]\right)}^{T}]$ and its mean square error $\Psi \left(\hat{\varrho }\right)=℧\left(\hat{\varrho }\right)+b\left(\hat{\varrho }\right)b^{T}\left(\hat{\varrho }\right)$ [28,32]. When RSS noise is sufficiently small, an estimator could be unbiased $b(\hat{\varrho })=0$ and $\Psi \left(\hat{\varrho }\right)=℧\left(\hat{\varrho }\right)$. In this case, the following inequality also holds

(18) $$\begin{array}{c} E\left[\left(\hat{\varrho }-E\left[\hat{\varrho }\right]\right){\left(\hat{\varrho }-E\left[\hat{\varrho }\right]\right)}^{T}\right]\geq \mathrm{Tr}\left\{\Gamma^{-1}\right\}, \end{array}$$

where **Γ** is the fisher information matrix (FIM) [32]. For multiple BMs, equivalent FIM (EFIM) is developed in [33] and has a form of

(19) $$\begin{array}{c} \Gamma \left(\varrho \right)=\left[\begin{array}{cc} \Upsilon & \Xi \\ \Xi^{T} & \Sigma \end{array}\right], \end{array}$$

where

(20) $$\begin{array}{c} \begin{array}{cc} \Upsilon & =-E\left[\frac{\partial^{2}L\left(x;\varrho \right)}{\partial \phi_{b}\partial \phi_{b}^{T}}\right],\\ \Xi & =-E\left[\frac{\partial^{2}L\left(x;\varrho \right)}{\partial \phi_{b}\partial \phi_{v}^{T}}\right],\\ \Sigma & =-E\left[\frac{\partial^{2}L\left(x;\varrho \right)}{\partial \phi_{v}\partial \phi_{v}^{T}}\right]. \end{array} \end{array}$$

By using a formula of partitioned matrix inversion similarly in [28], we can obtain

(21) $$\begin{array}{c} \mathrm{CRLB}\left(\phi_{b}\right)=\Upsilon^{-1}+\Upsilon^{-1}\Xi {\left(\Sigma -\Xi^{T}\Upsilon^{-1}\Xi \right)}^{-1}\Xi^{T}\Upsilon^{-1}. \end{array}$$

$\Upsilon^{-1}$ is similar to the CRLB of $\phi_{b}$ given in [34] when an exact value of $\phi_{v}$ is known. Consequently, the second term in Equation (21) is the cost in CRLB when $\phi_{v}$ is not known or known with errors. In [35], CRLB when $n_{b}=1$ is provided, which is a special case of Equation (21).

## 5. Numerical Results

**Figure 1:**
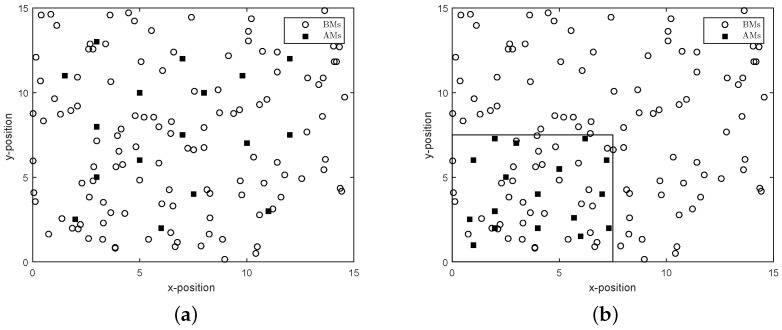
Distribution of anchor machines (AMs). (**a**) AMs uniformly distributed over the whole area; (**b**) AMs distributed uniformly in the lower left corner.

For numerical investigation, we assume 17 AMs, and 120 BMs are randomly distributed inside a square-region: {(x,y):0≤x≤S,0≤y≤S}, where S=15 unit of length. We use two different configurations of AM distribution: (a) uniformly distributed over the whole area (as shown in Figure 1a) and (b) uniformly distributed in the lower left corner (in Figure 1b). We assume a distance–power gradient of β=2, a standard deviation of measurement of $\sigma_{ij}=2$ dB and transmit power of $P_{j}=40$ dBm for all of the transmitting machines. We also assume that BMs can hear the signals from those AMs or VAMs, the actual distance to which is not greater than *R*, which is referred to as a *communication range*.

As a performance metric, we use a normalized RMSE

(22) $$\begin{array}{c} \mathrm{RMSE}=\frac{1}{Qn}\sum_{i=1}^{Q}\sum_{j=1}^{n}∥{\hat{\phi }}_{j}^{\left(i\right)}-\phi_{j}^{\left(i\right)}∥, \end{array}$$

where *Q* is the number of Monte Carlo runs, ${\hat{\phi }}_{j}^{\left(i\right)}$ is the estimated coordinate of BM *j* in the *i*-th run whose actual coordinate is $\phi_{j}^{\left(i\right)}$ and *n* is the total number of BMs. For each result in the figures below, Q=10,000 runs were done. The performance of proposed GSAP is compared with that of existing methods CL [7], WCL [8], LLS [10], subspace [11], and the CRLB provided in Equation (21). For a termination criterion of the localization algorithm, we have used $\delta =10^{-4}$.

**Figure 2:**
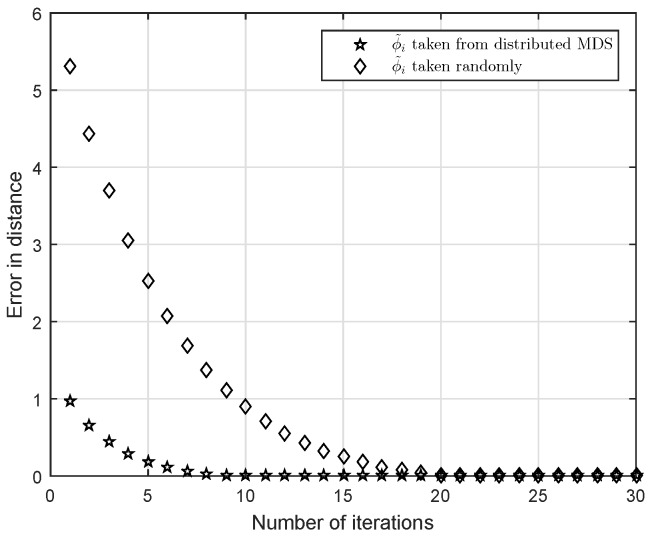
Estimate of blind machines (BM) vs. number of iterations using Equation (10).

We have suggested an algorithm of determining a good initial position of BMs using MDS. Figure 2 compares convergence speeds of the localization algorithm provided in this paper when a random initial position or a good initial position is used. For the test, an actual location of BM $\phi ={\left[4.0211,6.5256\right]}^{T}$ is investigated and *R* is assumed to be 2. A randomly generated initial point is $\tilde{\phi }={\left[0.40171,2.3843\right]}^{T}$ and an initial point obtained from MDS is $\tilde{\phi }={\left[3.3503,5.7309\right]}^{T}$. From Figure 2, we can see that the convergence is faster if the initial point from MDS is used. When we take $\delta =10^{-4}$ for a stopping criterion of the algorithm, a good initial point terminates the localization at nine iterations compared to 20 iterations with the random initial point. Both are convergent at the same position ${\left[4.0178,6.5206\right]}^{T}$.

**Figure 3:**
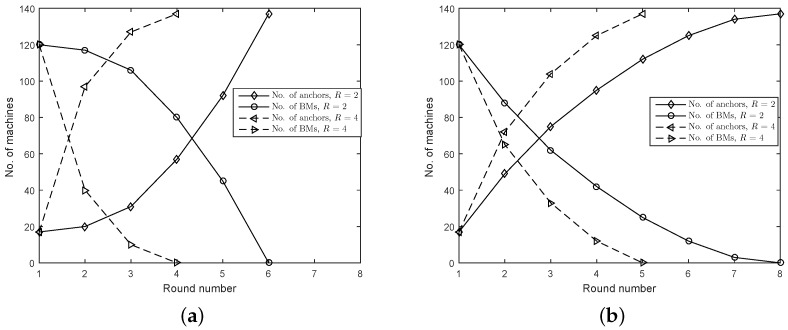
Number of rounds needed to localize the whole machine type communication (MTC) network. (**a**) AMs uniformly distributed over the whole area; (**b**) AMs distributed uniformly in the lower left corner.

In Figure 3a,b, the effect of AM distributions on the localization speed is investigated. They plot the number of machines marked as anchors (AMs or VAMs) and BMs at the start of each round in GSAP. In Figure 3a, at round 5, all of the 120 BMs can determine their locations when R=2. If R=4, only three rounds are enough. If AMs are located in the lower left corner, seven and four rounds are needed to determine the location of 120 BMs when R=2 and R=4, respectively. It is seen that a biased-distribution of AMs can severely degrade the localization speed, especially when the communication range is relatively small.

**Figure 4:**
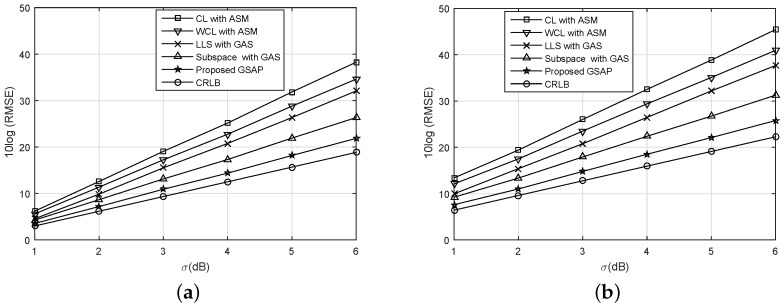
Root mean square error (RMSE) performance vs. $\sigma_{dB}$. (**a**) AMs uniformly distributed over the whole area; (**b**) AMs distributed uniformly in the lower left corner.

Figure 4 shows the effect of $\sigma_{dB}=\sigma_{ij}$ on the performance of localization algorithms. R=2 is assumed. In the simulation, identical $\sigma_{dB}$ is applied for each transmitter–receiver pair. For CL and WCL, anchor selection methods (ASM) provided in [20] are used, and the greedy anchor selection (GAS) proposed in this paper is used for LLS and Subspace techniques. The RMSE degrades (gets greater) as $\sigma_{dB}$ increases for every algorithm tested. The proposed GSAP, however, provides the best performance regardless of the assumptions on the AM distribution. Comparing the results from the two AM distributions, we can see that if AMs are uniformly distributed over the whole area, RMSE gain is about 4.165 dB compared with the locally distributed case at $\sigma_{dB}=3$. In both of the cases, the performance gap between GSAP and CRLB increases according to the increasing uncertainty.

**Figure 5:**
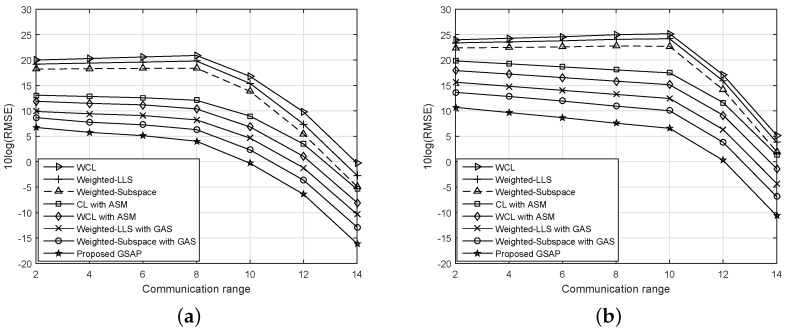
RMSE performance vs. communication range, R. (**a**) AMs uniformly distributed over the whole area; (**b**) AMs distributed uniformly in the lower left corner.

Figure 5 shows the effect of communication range *R* on the RMSE performance. When the communication range increases, each BM has the reference information from more anchors. However, since the location information from VAMs has some errors, the larger number of anchors is not always helpful. To make the comparison fair, we also have applied similar weight matrices, which are used in WCL, to the LLS and subspace techniques, referred to as weighted LLS and weighted subspace in Figure 5, respectively. It can be seen that, without anchor selection, the RMSE performance degrades though *R* increases in both Figure 5a,b. However, with anchor selection, it is improved with increasing *R*. If *R* is large enough (for example, when R≥10), most of the BMs can communicate with more than two anchors at the first round, and the RMSE can be reduced though anchor selection is not used. Comparing the performances with and without anchor selection, the localization algorithms with certain anchor selection certainly achieve better RMSE than without the anchor selection if the communication range is limited.

## 6. Conclusions

In this paper, we have proposed and investigated GSAP. In an MTC network that consists of a certain number of AMs and many BMs, GSAP eventually estimates the location of all the BMs. Though some BMs cannot find enough anchors in their respective neighbors in an initial round of GSAP, they can find anchors in the following rounds by regarding positioned BMs as VAMs. In this procedure, RMSE is, of course, increasing by the positioning error in VAMs, but it is numerically shown that GSAP provides RMSE better than the existing methods (CL, WCL, LLS and subspace techniques). Specifically, the proposed method achieves about 52%–97% improvement in terms of RMSE when AMs are uniformly distributed over the area. If AMs are located in a corner, the improvement increases to about 57%–98%. To reduce the effect of positioning error in VAMs, GSAP selects and uses only the three best anchors in terms of the errors. The RMSE performance of GSAP is also shown to be close to the CRLB. GSAP is a generic procedure in finding the location of BMs in a relatively large area with a limited number of anchors and is promising in determining the location of MTC devices in future Internet of Things applications.

## Figures and Tables

**Table 1:** Symbols and notations. AMs: anchor machines; BMs: blind machines; VAMs: virtual anchor machines.

Symbol	Description
*N*	Total number of AMs and BMs
*n*	Total number of BMs at the start of localization process
*m*	Total number of AMs
$n_{v}$	Total number of VAMs
$n_{b}=n-n_{v}$	Total number of BMs
$a=m+n_{v}$	Total number of anchors (i.e., AMs plus VAMs)
ϕ	Actual position vector of machines
$\phi_{b}$	Actual position vector of BMs
$\phi_{v}$	Actual position vector of VAMs
${\hat{\phi }}_{v}$	Estimated position of VAMs
$S_{A}$	Set of AMs
$S_{V}$	Set of VAMs
$S_{U}$	Set of BMs
$S_{S}$	Set of selected anchors who will participate in localization
$\vartheta^{p}$	Expected position error (scalar)
$\vartheta^{r}$	Expected error in measurement (scalar)
*P*	Transmitted power (scalar)
*ς*	Power loss in dB (scalar)
*η*	RSS noise (scalar)
*β*	Distance-power gradient (scalar)
*d*	Actual distance (scalar)
$\tilde{d}$	Observed range (scalar)
*ζ*	Weight for anchors based on $\vartheta^{p}$ and $\vartheta^{r}$ which is used in anchor selection (scalar)
p	Observed path loss in dB (vector)
**Π**	Jacobian matrix
$\tilde{\phi }$	Initial position vector for BMs
R	Wieghting matrix used in weighted least square formulation
${\tilde{\phi }}^{*}$	Final estimated position vector for BMs
D	Proximity information matrix
**Ω**	Double centered matrix
***ϱ***	Actual position vector of all BMs
$Q_{\phi_{v}}$	Covariance matrix for error in ${\hat{\phi }}_{v}$ which we assume a white Gaussian process
b	Bias of an estimator (vector)
℧	Covariance matrix of an estimator
**Ψ**	Mean square error matrix
E	Expectation operator
**Γ**	Fisher information matrix
$X_{D}\left(.\right)$	Stress function used in multidimensional scaling
L(.)	Log-likelihood function
g(.)	Probability density function
${\left(.\right)}^{-1}$	Inverse of a matrix
${\left(.\right)}^{T}$	Transpose of a vector or matrix
Tr(.)	Trace of a matrix

## Abbreviations

The following abbreviations are used in this manuscript:

MTC	Machine type communication
AM	Anchor machine
BM	Blind machine
CRLB	Cramér–Rao lower bound
GPS	Global positioning system
CL	Centroid localization
WCL	Weighted centroid localization
RSS	Received signal strength
VAM	Virtual anchor machine
SAP	Successive *anchorization* process
GSAP	Greedy successive *anchorization* process
MDS	Multidimensional scaling
LLS	Linear least square
RMSE	Root mean square error
PIM	Proximity information matrix
FIM	Fisher information matrix
ASM	Anchor selection method
GAS	Greedy anchor selection
